# β_1_-Blockers Lower Norepinephrine Release by Inhibiting Presynaptic, Facilitating β_1_-Adrenoceptors in Normotensive and Hypertensive Rats

**DOI:** 10.3389/fneur.2014.00051

**Published:** 2014-04-16

**Authors:** Torill Berg

**Affiliations:** ^1^Department of Physiology, Institute of Basic Medical Sciences, University of Oslo, Oslo, Norway

**Keywords:** β-adrenoceptors, atenolol, metoprolol, norepinephrine release, adrenal glands, angiotensin II, hypertension

## Abstract

Peripheral norepinephrine release is facilitated by presynaptic β-adrenoceptors, believed to involve the β_2_-subtype exclusively. However, β_1_-selective blockers are the most commonly used β-blockers in hypertension. Here the author tested the hypothesis that β_1_AR may function as presynaptic, release-facilitating auto-receptors. Since β_1_AR-blockers are injected during myocardial infarction, their influence on the cardiovascular response to acute norepinephrine release was also studied. By a newly established method, using tyramine-stimulated release through the norepinephrine transporter (NET), presynaptic control of catecholamine release was studied in normotensive and spontaneously hypertensive rats. β_1_AR-selective antagonists (CGP20712A, atenolol, metoprolol) reduced norepinephrine overflow to plasma equally efficient as β_2_AR-selective (ICI-118551) and β_1+2_AR (nadolol) antagonists in both strains. Neither antagonist lowered epinephrine secretion. Atenolol, which does not cross the blood–brain barrier, reduced norepinephrine overflow after adrenalectomy (AdrX), AdrX + ganglion blockade, losartan, or nephrectomy. Atenolol and metoprolol reduced resting cardiac work load. During tyramine-stimulated norepinephrine release, they had little effect on work load, and increased the transient rise in total peripheral vascular resistance, particularly atenolol when combined with losartan. In conclusion, β_1_AR, like β_2_AR, stimulated norepinephrine but not epinephrine release, independent of adrenal catecholamines, ganglion transmission, or renal renin release/angiotensin AT1 receptor activation. β_1_AR therefore functioned as a peripheral, presynaptic, facilitating auto-receptor. Like tyramine, hypoxia may induce NET-mediated release. Augmented tyramine-induced vasoconstriction, as observed after injection of β_1_AR-blocker, particularly atenolol combined with losartan, may hamper organ perfusion, and may have clinical relevance in hypoxic conditions such as myocardial infarction.

## Introduction

Beta-blockers have been first-line drugs in the treatment of hypertension for half a century. The β-adrenoceptors (AR) comprise the β_1_-_,_ β_2_-, and β_3_-subtypes. Today, the β_1_-selective antagonist atenolol is the beta-blocker most frequently used in hypertension and is also recommended as preventive medication after myocardial infarction. Metoprolol, another β_1_AR-selective antagonist, which unlike atenolol crosses the blood–brain barrier, is commonly used also during acute myocardial infarction.

**Figure 1:**
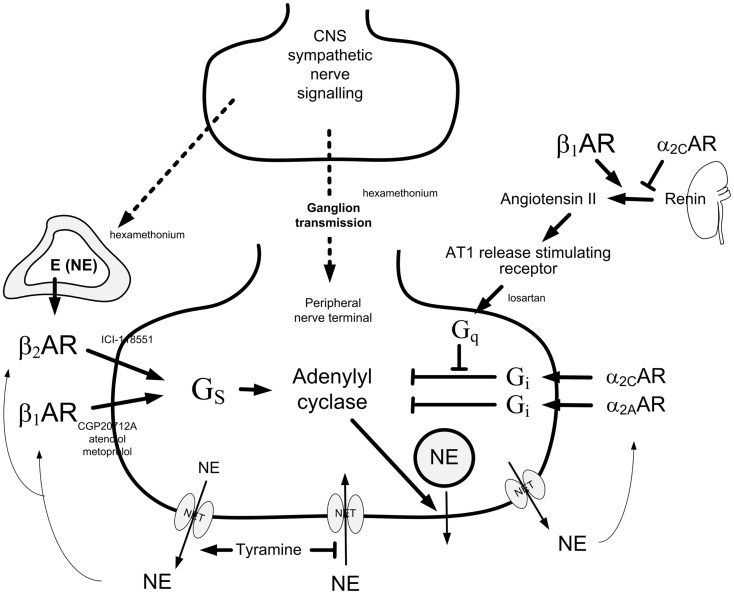
**Presynaptic control of norepinephrine release from peripheral sympathetic nerve endings**. Presynaptic modulation of vesicular release is reflected as differences in overflow to plasma only when re-uptake through NET is blocked (9). Tyramine stimulates norepinephrine release from peripheral sympathetic nerve terminals by reverse transport through NET. Consequently, re-uptake through NET is prevented. The influence of presynaptic release control can therefore be studied by differences in the tyramine-induced norepinephrine overflow to plasma (10). The presynaptic receptors will be activated by the released transmitters, or by other agonists present in their vicinity. The release of norepinephrine from secretory granules is activated by adenylyl cyclase, which is stimulated and inhibited, respectively, by stimulatory (Gs) and inhibitory (Gi) G proteins. Gs- and Gi-activation is mediated by β_1+2_AR and α_2_AR presynaptic receptors, respectively. The AT1R augment adenylyl cyclase activity by inhibiting Gi-signaling. β_1_AR and α_2_AR also modulate renin release from the kidneys. The nicotinic receptor antagonist hexamethonium will inhibit ganglion transmission as well as nerve-stimulated epinephrine release from the adrenals. Dotted arrows indicate nerve signals, curved arrows indicate action of tyramine-released norepinephrine, thick arrows indicate positive effects, whereas blunted arrows indicate inhibitory actions. Modified from Ref. (11).

Release of norepinephrine from peripheral sympathetic nerve terminal vesicles is stimulated or inhibited by presynaptic receptors (Figure 1). The first description of release-stimulating AR was made by Adler-Graschinsky and Langer (1): β_2_AR but not β_1_AR agonists dose-dependently increased nerve-stimulated norepinephrine release, and the stimulating effect of isoproterenol on release was blocked by β_2_AR but not β_1_AR antagonists (2–4). It has therefore been accepted that the presynaptic, release-stimulating βAR is of the β_2_-subtype exclusively. Since β_2_AR are by far more effectively activated by epinephrine than norepinephrine, it has been suggested that they are activated by circulating epinephrine, or epinephrine co-released with norepinephrine after re-uptake through the norepinephrine transporter (NET) (5).

However, we previously observed in whole-animal experiments that not only the β_2_AR-selective antagonist ICI-118551 but also the β_1_AR-selective antagonist CGP20712A reduced tyramine-stimulated norepinephrine release in both spontaneously hypertensive (SHR) and normotensive (WKY) rats (6). These reductions may result from a direct effect on presynaptic β_1_AR, facilitating release of norepinephrine and epinephrine. However, CPG20712A may cross the blood–brain barrier (7), and its effect could therefore have resulted from a central action, thus altering sympathetic nerve and/or adrenal release. Furthermore, intrathoracic ganglion transmission has been shown to be facilitated by both β_1_AR and β_2_AR (8). It is therefore possible that CPG20712A reduced norepinephrine overflow either directly by hampering ganglion transmission, or indirectly by lowering intrathoracic ganglion transmission, splanchnic nerve activation, adrenal epinephrine secretion and, hence, β_2_AR-mediated stimulation of norepinephrine release. β_1_AR in addition stimulate renal renin secretion (12), which, through subsequent activation of presynaptic angiotensin AT1 receptors (AT1R), may facilitate norepinephrine release (13). Multiple mechanisms other than presynaptic modulation may therefore be responsible for the CPG20712A-induced reduction in norepinephrine release.

Norepinephrine and epinephrine have direct effects on cardiac and vascular postsynaptic AR. Vascular β_1_AR may either cause vasodilatation by activating vascular smooth muscle cell (VSM) β_1_AR, or vasoconstriction by activating endothelial β_1_AR and inhibiting endothelium-dependent hyperpolarization (14). β_1_AR antagonists may therefore influence heart function and organ blood flow directly, independent of their possible effects on catecholamine release.

The purpose of the present investigation was therefore to test the hypothesis that β_1_AR-mediated stimulation of norepinephrine release involved peripheral, presynaptic auto-receptors, or alternatively, was due to epinephrine secretion, reduced ganglion transmission, or reduced renal renin secretion with subsequent presynaptic AT1R-activation. The second purpose was to study how βAR antagonists influenced total peripheral vascular resistance (TPR), cardiac function, and blood pressure (BP) at rest and during stimulated norepinephrine release. Owing to the extensive use of β_1_-selective blockers in the treatment of hypertension and myocardia infarction, the last goal was to test if β_1_AR-functions differed in WKY and SHR.

**Figure 2:**
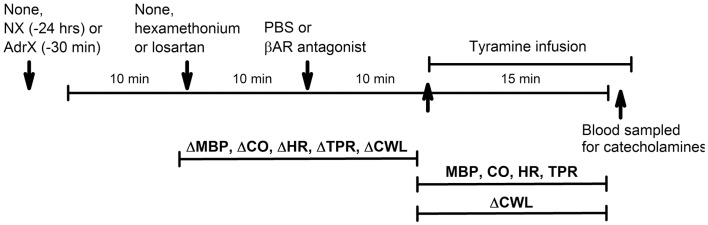
**Outline of the experimental design**. During pre-treatment, the rats were subjected to (arrows) (1) none or surgical intervention with NX or AdrX, (2) no injection or injection of PBS or hexamethonium or losartan, and (3) injection of PBS or βAR antagonist, in combinations as outlined in Table 1. All rats were subsequently infused with tyramine. Changes in MBP, CO, HR TPR, and cardiac work load (CWL) were recorded from before drug pre-treatment to before tyramine, and every min throughout the tyramine infusion for MBP, CO, HR, and TPR, and from before to the end of the 15-min tyramine infusion for CWL. The effect of NX and AdrX was analyzed by comparing the baselines prior to drug pre-treatment with that in rats not subjected to surgical intervention. Blood for measurement of plasma catecholamines was collected from the femoral artery after the 15-min tyramine-observation period but without discontinuing the infusion.

**Table 1:** **The plasma concentration of norepinephrine and epinephrine**.

	WKY			SHR		
	*N*	Norepinephrine (nM)	Epinephrine (nM)	*N*	Norepinephrine (nM)	Epinephrine (nM)
**PROTOCOL 1: ROLE OF β_1+2_AR IN CATECHOLAMINE RELEASE**						
PBS + tyramine	13	21.7 ± 0.9	3.8 ± 1.0	16	26.7 ± 1.3**	5.7 ± 0.7
Atenolol + tyramine (peripheral β_1_AR ant.)	7	11.6 ± 0.8^‡‡^	2.6 ± 0.4	8	13.8 ± 1.1^‡‡^	7.9 ± 3.0
CGP20712A + tyramine (β_1_AR ant.)	7	11.4 ± 1.0^‡‡^	2.9 ± 0.8	7	16.7 ± 2.4^‡‡^	7.2 ± 3.5
Metoprolol + tyramine (β_1_AR ant.)	6	9.3 ± 0.7^‡‡^	1.8 ± 0.7	6	14.6 ± 1.2^‡‡^	13.4 ± 4.5
ICI-118551 + tyramine (β_2_AR ant.)	6	16.3 ± 1.9^‡^	3.2 ± 1.5	7	15.0 ± 1.4^‡‡^	5.4 ± 1.1
Nadolol + tyramine (peripheral β_1+2_AR ant.)	7	10.2 ± 2.5^‡‡^	1.8 ± 1.0	6	13.1 ± 1.9^‡‡^	7.4 ± 2.9
**PROTOCOL 2: ROLE OF THE ADRENALS IN β_1/2_AR MODULATION OF NOREPINEPHRINE RELEASE**						
AdrX + PBS + tyramine	7	23.1 ± 2.5	0.6 ± 0.6^§§^	8	31.8 ± 3.1	0.2 ± 0.1^§§^
AdrX + atenolol + tyramine	6	9.8 ± 0.4^‡‡^	0.0 ± 0.0	8	23.0 ± 2.4^‡^	0.0 ± 0.0
AdrX + CGP20712A + tyramine	7	15.2 ± 4.9^‡^	0.0 ± 0.0	7	22.5 ± 2.0^‡^	0.1 ± 0.0
AdrX + ICI-118551 + tyramine	7	15.3 ± 1.6^‡^	0.0 ± 0.0	9	22.7 ± 2.5^‡^	0.4 ± 0.2
AdrX + nadolol + tyramine	8	11.6 ± 1.9^‡‡^	0.0 ± 0.0	11	21.0 ± 2.8^‡^	0.2 ± 0.2
**PROTOCOL 3: ROLE OF ADRENAL NICOTINIC RECEPTORS AND GANGLION TRANSMISSION IN β_1_AR MODULATION OF CATECHOLAMINE RELEASE**						
Hexamethonium + tyramine (nicotinic receptor ant.)	6	26.1 ± 1.4^†^	0.6 ± 0.3^†^	6	19.6 ± 0.9^††^	1.7 ± 0.5^††^
AdrX + hexamethonium + tyramine	6	23.0 ± 1.3	0.4 ± 0.3	9	62.1 ± 9.0^§§^	0.6 ± 0.4
AdrX + hexamethonium + atenolol + tyramine	6	12.5 ± 0.6^‡‡^	0.6 ± 0.6	6	37.9 ± 5.0^‡^	0.6 ± 0.6
**PROTOCOL 4: ROLE OF THE RENIN-ANGIOTENSIN SYSTEM AND THE KIDNEYS IN β_1_AR MODULATION OF CATECHOLAMINE RELEASE**						
Losartan + tyramine (angiotensin AT1 receptor ant.)	9^a^	18.4 ± 0.7^†^	4.2 ± 1.5	6^a^	28.4 ± 3.4	11.8 ± 4.1
Losartan + atenolol + tyramine	6	12.4 ± 0.9^‡‡^	1.4 ± 0.2	6	17.1 ± 1.7^‡^	7.1 ± 1.6
Losartan + metoprolol + tyramine	6	10.5 ± 0.5^‡‡^	2.8 ± 0.5	6	15.1 ± 1.5^‡^	11.9 ± 2.6
AdrX + losartan + tyramine	7	19.3 ± 2.0	0.2 ± 0.1^§^	12	35.6 ± 4.7	0.2 ± 0.0^§§^
AdrX + losartan + atenolol + tyramine	7	13.4 ± 0.7^‡^	0.7 ± 0.7^§^	6	47.5 ± 6.0^§§^	0.0 ± 0.0^§§^
NX + PBS + tyramine	7	33.9 ± 1.6^††^	16.1 ± 4.0^†^	6	34.2 ± 2.5^†^	8.2 ± 1.1
NX + atenolol + tyramine	6	24.6 ± 1.4^‡‡^	4.7 ± 1.0^‡^	6	25.2 ± 2.9^‡^	7.0 ± 1.4

Comparisons were made between the SHR and WKY control groups (* after SHR values). Within each strain, comparisons were made between the PBS + tyramine control groups and the groups pre-treated with hexamethonium, losartan, or NX alone (^†^), between corresponding groups without or with pre-treatment with βAR antagonist (^‡^) and corresponding groups without or with AdrX (§). Differences were detected as indicated.

*N, number of rats per group. Ant., antagonist*.

^a^From Ref. (11). ^†^, ^‡^, § – *P * ≤ 0.05, **, ^††^, ^‡‡^, §§ – *P * ≤ 0.005.

## Materials and Methods

### Experimental procedure

#### Animals

All experiments were approved by The Norwegian Animal Research Authority (NARA) and conducted in accordance with the Directive 2010/63/EU of the European Parliament. 212 male, 12–14 weeks old SHR (Okamoto, SHR/NHsd strain, 279 ± 2 g body weight) and 168 WKY (Wistar Kyoto, 281 ± 2 g body weight) on conventional rat chow diet (0.7% NaCl) were included in the study.

#### Anesthesia

The rats were anesthetized with pentobarbital (65–75 mg/kg, IP). The level of surgical anesthesia was tested by non-responsiveness to pinching between the toes. When satisfactory anesthesia was established, it remained throughout the experimental period without further supply.

#### Surgical procedure

The rats were tracheotomized, and a heparinized catheter was inserted into the femoral artery to record systolic (SBP) and diastolic (DBP) BP. The rats were then connected to a positive-pressure respirator. Entering the thoracic cavity through the third intercostal space, a 2SB perivascular flow probe, connected to a T206 Ultrasonic Transit-Time Flowmeter (Transonic Systems Inc., Ithaca, NY, USA), was placed on the ascending aorta to measure cardiac output (CO, i.e., without cardiac flow) and heart rate (HR). The thoracic cavity was subsequently closed with a suture, but the rats remained on the respirator and were ventilated with air throughout the experiment. A stabilizing period of 10 min was allowed before the first experimental drug was injected. Mean arterial BP (MBP = (SBP − DBP)/3 + DBP), TPR (MBP/CO), and cardiac work (SBP×HR) were calculated. Body temperature was maintained at 37–38°C by external heating, guided by a thermo sensor inserted inguinally into the abdominal cavity. Bilateral nephrectomy (NX) or adrenalectomy (AdrX) was performed through flank incisions. NX was done 24 h prior to the experiment during short-time anesthesia [fentanyl citrate (0.16 mg/kg)/fluanisone (5 mg/kg)/midazolam (2.5 mg/kg)], and AdrX at the start of the surgical preparation for the experiment, i.e., 30 min before injecting the first drug. The arterial catheter was flushed with 0.15 mL buffered saline (PBS; 0.01 M Na-phosphate, pH 7.4, 0.14 M NaCl) containing 500 IU/mL heparin. Drugs were dissolved in PBS and administered as bolus injections through a catheter in the femoral vein (0.6–1.0 mL/kg), unless otherwise indicated. When the experiment was completed, the rats were sacrificed by an IV injection of about 35 mg pentobarbital.

### Working hypotheses (Figure 1) and experimental protocols (Figure 2)

#### Protocol 1: does β_1_AR, like β_2_AR, stimulate catecholamine release?

To answer this question, the rats were pre-treated with βAR antagonists, and 10 min later infused for 15 min with tyramine (1.26 μmol/kg/min). Tyramine stimulates norepinephrine release from peripheral sympathetic nerve endings by activating reverse transport through NET (15). Thus, by engaging NET in release, re-uptake is prevented, and presynaptic modulation of vesicular release is reflected as differences in the overflow of norepinephrine to plasma (Figure 1), as previously documented in detail (9, 10). The following βAR antagonists were tested: the peripherally restricted, i.e., which does not cross the blood–brain barrier, β_1_AR-selective antagonist atenolol (5.6 μmol/kg), the not restricted metoprolol (8.8 μmol/kg, β_1_) or CGP20712A (11 μmol/kg, β_1_), the β_2_AR-selective antagonist ICI-118551 (initial dose of 1 μmol/kg, followed by 0.3 μmol/kg/min throughout the experiment), or the peripherally restricted, β_1+2_AR antagonist nadolol (8.5 μmol/kg) (6).

#### Protocol 2: is the hampering effect of βAR antagonists on norepinephrine overflow to plasma dependant on adrenal secretion of epinephrine?

To test if the reduced norepinephrine overflow after pre-treatment with βAR antagonists depended on secretion of epinephrine from the adrenals, AdrX rats were pre-treated with atenolol, CGP20712A, ICI-118551, or nadolol, followed by tyramine, as above.

#### Protocol 3: does the hampering effect of β_1_AR antagonists on norepinephrine overflow to plasma involve inhibition of ganglion transmission?

In this protocol, the author tested if the β_1_AR antagonist-induced reduction in norepinephrine overflow was due to a β_1_AR-mediated inhibition of ganglion transmission in AdrX rats. The rats were therefore pre-treated with the ganglion blocker, nicotinic receptor antagonist hexamethonium (37 μmol/kg) (16), and subsequently infused with tyramine as above. Since also not AdrX rats were pre-treated with hexamethonium, this protocol in addition tested if adrenal epinephrine secretion was mediated through adrenal nicotinic receptors.

#### Protocol 4: is the hampering effect of β_1_AR antagonists on norepinephrine overflow due to inhibition of renal renin release and, through that, inhibition of presynaptic, release-facilitating angiotensin AT1 receptors?

The rats were pre-treated with the AT1R antagonist losartan (79 μmol/kg) (16), alone, or followed 10 min later by atenolol or metoprolol, as above. Ten minutes later, all rats were infused with tyramine. To control for a possible central effect of losartan with subsequent reduced stress-induced sympathoadrenal activation (17), AdrX rats were pre-treated with losartan, alone or combined with atenolol, as above. PBS or atenolol followed by tyramine were also given to 24-h NX rats, where a possible influence from renal renin secretion will be eliminated.

In all protocols, control rats were pre-treated with PBS, and included randomly throughout all protocols. Except for the WKY and SHR control groups which comprised 13 and 16 rats, respectively, each group comprised 6–9 rats (Table 1), with >6 rats included if the intragroup variation in the catecholamine data was large or to ensure that experiments within all four protocols overlapped in time. The cardiovascular analyses included data from additional rats, where catecholamine measurements were compromised or the catecholamines determined by a different method. This concerned primarily the AdrX+PBS−, hexamethonium- and losartan-pre-treated groups, which included 10–13 rats per group.

### Measurement of plasma catecholamines

1.5 mL blood was collected by free flow from the femoral artery into tubes containing 45 μl 0.2 M glutathione, 0.2 M EGTA (4°C). Plasma was stored at −80°C until catecholamine concentrations were determined using 400 μl plasma and the 5000 Reagent kit for HPLC analysis of Catecholamines in plasma from Chromsystems GmbH, Munich, Germany, as described by the manufacturer.

### Statistical analyses

All results are presented as mean values ±SE mean. Since the results in the control groups were found to remain consistent throughout the study, these rats were pooled into one WKY and one SHR control group.

The plasma catecholamine concentrations were evaluated by one-way ANOVA, first including all groups within each strain, then including groups within each of the four protocols, or including the controls, AdrX + PBS-, hexamethonium-, losartan-, and NX-pre-treated groups. When the presence of group differences was indicated, these were subsequently located by two-sample Student’s *t*-tests for parametric data, and Kruskal–Wallis tests for non-parametric results. *P * ≤ 0.05 was considered significant.

The cardiovascular data were averaged every min. The cardiovascular response to pre-treatment and baselines prior to tyramine were evaluated by one-way ANOVA, first including, within each strain, all groups indicated in Table 2, then for sets of experiments. When the presence of group differences was indicated, these were subsequently located by two-sample Student’s *t*-tests for parametric data, and Kruskal–Wallis tests for non-parametric results. Each step and each test employed Bonferroni-adjusted *P*-values.

**Table 2:** **The effect of pre-treatment on cardiac work load (SBP × HR) during rest and during the response to tyramine**.

Pre-treatment	WKY		SHR	
	Pre-treatment (%)	Tyramine 15 min (%)	Pre-treatment (%)	Tyramine 15 min (%)
PBS	−4.0 ± 3.7	168.9 ± 17.5	−1.4 ± 6.1	110.7 ± 9.3
Atenolol	−19.2 ± 2.3^‡‡^	129.0 ± 5.5	−38.5 ± 5.0^‡‡^	115.0 ± 14.1
Metoprolol	−12.9 ± 5.5	142.1 ± 24.2	−26.1 ± 9.1	120.5 ± 16.8
PBS after AdrX	−6.7 ± 3.9	191.8 ± 14.8	−13.9 ± 3.7	84.9 ± 14.6
Atenolol after AdrX	−16.5 ± 6.6	105.1 ± 11.7^‡‡^	−34.2 ± 2.6^‡‡^	52.0 ± 10.9
Hexamethonium	−29.4 ± 3.3^††^	218.5 ± 15.1^†^	−50.2 ± 6.8^††^	242.4 ± 22.8^††^
Hexamethonium after AdrX	−17.7 ± 1.5^††^	200.6 ± 15.1^†^	−52.8 ± 2.8^††^	118.3 ± 29.6
Hexamethonium + atenolol after AdrX	−25.8 ± 6.0	140.0 ± 10.8^‡‡^	−52.6 ± 4.0	62.3 ± 7.7
Losartan^a^	−27.8 ± 3.1^††^	158.0 ± 16.3	−25.6 ± 7.6	150.4 ± 15.7
Losartan + atenolol	−29.0 ± 5.5	163.4 ± 14.9	−46.3 ± 4.2^‡^	166.9 ± 22.0
Losartan + metoprolol	−24.4 ± 7.0	138.3 ±8.2	−58.2 ± 2.3^‡‡^	225.7 ± 19.7^‡^
Losartan after AdrX	−34.1 ± 2.9^††^	167.5 ± 11.3	−38.9 ± 3.6^††^	105.4 ± 17.2
Losartan + atenolol after AdrX	−38.6 ± 5.8	180.3 ± 33.4	−54.0 ± 3.2^‡‡^	59.8 ± 19.3
PBS after NX	−21.1 ± 5.1^†^	105.1 ± 14.7^†^	−11.6 ± 4.2	100.2 ± 11.9
Atenolol after NX	−8.0 ± 3.7	86.5 ± 7.8	−10.7 ± 3.0	67.8 ± 8.7

*The changes in cardiac work load from before to after pre-treatment were expressed in percentage of the load prior to pre-treatment, and that in response to tyramine as change from before to that after the 15-min infusion in percentage of the before value. Comparisons were made within each strain between the PBS + tyramine control groups and the groups pre-treated with AdrX, hexamethonium, AdrX + hexamethonium, losartan, AdrX + losartan or NX without β_1_AR antagonist ( ^†^), and between corresponding groups without or with pre-treatment with β_1_AR antagonist ( ^‡^)*.

*^a^From Ref. (11). Differences were detected as indicated. ^†^, ^‡^ – *P * ≤ 0.05, ^††^, ^‡‡^ – *P * ≤ 0.005*.

Owing to differences in baselines, changes in the cardiovascular responses to tyramine were presented in percentage of baselines. The tyramine response-curves were analyzed using Repeated Measures Analyses of Variance and Covariance, first as overall tests within each strain including all groups or sets of groups, and subsequently between groups or for each group separately. Significant responses (one-sample Student’s *t*-tests) and groups differences (two-sample Student’s *t*- or Kruskal–Wallis tests) were subsequently located at specific times, i.e., at the initial peak-pressure response (about 3 min) and/or after 15 min. Testing proceeded only when the presence of significant responses, differences and/or interactions was indicated. The *P*-value was for all tests and each step adjusted according to Bonferroni.

Differences in the cardiac work load in response to pre-treatment or tyramine was analyzed by one-way ANOVA, followed by two-sample Student’s *t*- or Kruskal–Wallis tests as above. *P * ≤ 0.05 was considered significant.

### Drugs

Losartan was a kind gift from Merck, Sharp and Dohme Labs, Rahway, NJ, USA. ICI-118551 was obtained from ICI-Pharma, Cheshire, UK, metoprolol tartrate from Tocris Bioscience, Bristol, UK, fentanyl citrate/fluanisone (Hypnorm) from VetaPharma Ltd., Leeds, UK and midazolam from Actavis Norway, Oslo, Norway. Pentobarbital was from The Norwegian National Hospital, Oslo, Norway. The remaining drugs were from Sigma Chemical Co., St. Louis, MO, USA.

## Results

### Role of β_1+2_AR in tyramine-induced norepinephrine overflow to plasma

The tyramine-induced overflow of norepinephrine to plasma was higher in the SHR than in the WKY controls (*P * = 0.003) (Table 1). This overflow was reduced after pre-treatment with atenolol (β_1_), CGP20712A (β_1_), metoprolol (β_1_), ICI-118551 (β_2_), and nadolol (β_1+2_) in both strains. In WKY, the three β_1_AR antagonists reduced the plasma concentration to <43% of that in the controls, whereas the effect of the β_2_AR-selective ICI-118551 was less (*P * ≤ 0.046), i.e., reduced to 73%. A similar difference between β_1_- and β_2_AR antagonists was not observed in SHR. The effect of the β_1_- and β_2_AR antagonists was not additive (*P * = NS for the nadolol compared to the β_1 or 2_AR-blocker groups).

The plasma norepinephrine concentration in AdrX rats was not different from that in the controls, and the lowering effect of βAR antagonists on the tyramine-induced norepinephrine overflow remained in both strains. These results showed that β_1/2_AR stimulation of norepinephrine release did not depend on circulating catecholamines secreted by the adrenals during the experiment.

The nicotinic receptor antagonist and ganglion blocker hexamethonium slightly increased the plasma norepinephrine concentration in WKY (*P * = 0.028), but decreased the concentration in SHR (*P * < 0.001). To eliminate possible indirect effects due to inhibition of adrenal nicotinic receptors and epinephrine secretion, this experiment was repeated in AdrX rats. The effect of hexamethonium was not different in AdrX WKY, whereas hexamethonium increased overflow in AdrX SHR (*P * ≤ 0.011 compared to the PBS- or AdrX + PBS-pre-treated SHR groups). However, additional pre-treatment with atenolol reduced norepinephrine overflow in both strains (*P * ≤ 0.039).

The AT1R antagonist losartan slightly reduced tyramine-induced norepinephrine overflow in WKY (*P * = 0.011), but not in SHR. Pre-treatment with losartan + atenolol reduced the plasma concentration in WKY, AdrX WKY and in SHR (*P * ≤ 0.016 compared to that after losartan alone), but not in AdrX SHR (*P * = NS). Also metoprolol reduced the plasma norepinephrine concentration in losartan-treated WKY and SHR (*P * = NS compared to metoprolol alone, and *P * ≤ 0.013 compared to losartan alone). Tyramine-induced norepinephrine overflow was higher in NX rats of both strains (*P * ≤ 0.015), but atenolol reduced the tyramine-induced norepinephrine overflow also after NX (*P * ≤ 0.037).

### Role of β_1/2_AR in modulating secretion of epinephrine

The plasma concentration of epinephrine in the SHR controls was not different from that in the WKY controls (*P * = NS) (Table 1). Epinephrine was almost totally absent in plasma from AdrX rats, and was clearly reduced in hexamethonium-treated rats (*P * ≤ 0.009), whereas losartan had no effect. The epinephrine concentration was not altered after pre-treatment with βAR antagonists, atenolol combined with hexamethonium or losartan, or metoprolol combined with losartan. The concentration of epinephrine was higher after NX in WKY (*P * = 0.007) but not in SHR, and was reduced by atenolol in NX rats of both strains (*P * ≤ 0.022).

### Cardiovascular response to pre-treatment

In accordance with previous studies, MBP, HR, TPR, and cardiac work load at the start of the experiment were higher in SHR than in WKY (96 ± 12 and 68 ± 4 mm Hg in SHR and WKY, respectively, 415 ± 8 and 343 ± 9 bpm, 5.3 ± 0.3 and 2.5 ± 0.2 mm Hg/mL/min, and 47,049 ± 3335 and 28,625 ± 1852 mm Hg × bpm), whereas CO was less (18 ± 1 and 28 ± 2 mL/min) (*P * < 0.001). These parameters were clearly less in hexamethonium-treated SHR (data not shown, Table 2 for cardiac work load). The present analyses focused mainly on the effect of atenolol and metoprolol. In short, atenolol reduced baseline HR (ΔHR = −37 ± 6 and −71 ± 12 bpm in WKY and SHR, respectively, *P * ≤ 0.004 compared to the PBS-sham-injection in the controls) and in SHR also MBP (89 ± 6 mm Hg in the controls and 64 ± 2 mm Hg after atenolol) and CO (19 ± 1 in the controls and 12 ± 1 mL/min after atenolol) (*P* ≤ 0.005). Metoprolol reduced HR in SHR only (ΔHR = −65 ± 10 bpm, *P * = 0.004), but had no significant effect on MBP. Atenolol reduced the cardiac work load (*P * ≤ 0.003) with a greater effect (*P * ≤ 0.007) in SHR than in WKY, whereas metoprolol had no effect (*P * = NS) (Table 2).

### Cardiovascular response to tyramine

**Figure 3:**
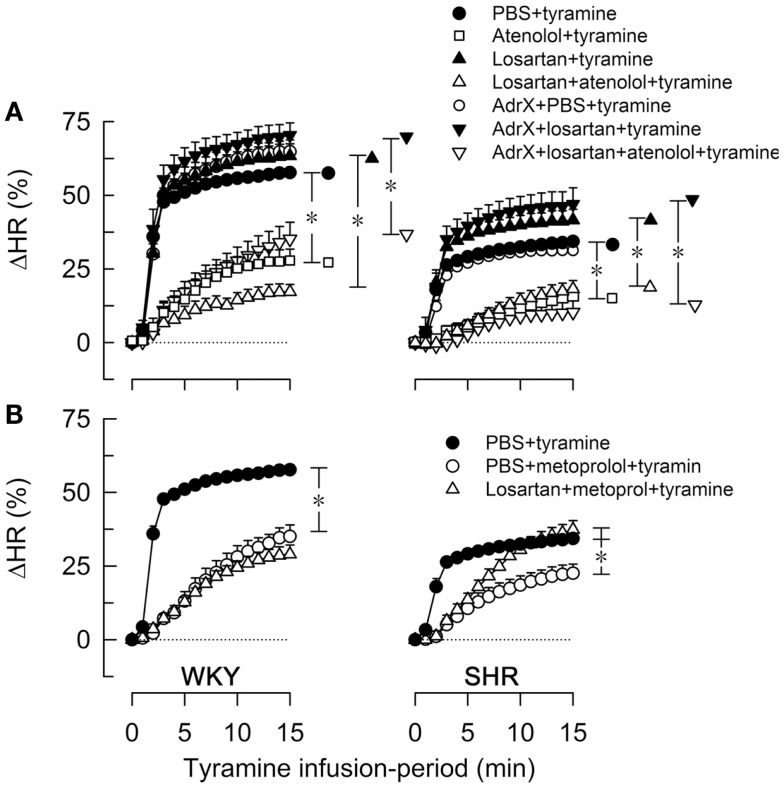
**The effect of atenolol (A) or metoprolol (B) on the HR-response to tyramine in WKY and SHR**. The rats were pre-treated as outlined in the legends. After overall and step-by-step curve evaluations with Repeated Measures Analyses of Variance and Covariance (please see Statistical Analyses), group differences were located at 15 min (* in brackets right of curves), as indicated. Comparisons were made in **(A)** between the controls and losartan-treated groups, between the losartan- and AdrX + losartan-pre-treated groups and between corresponding groups without and with pre-treatment with atenolol; and in **(B)** between the controls and the metoprolol-treated groups, and between the metoprolol- and the losartan + metoprolol-treated groups. In the same order as in the legends, HR baselines prior to tyramine was in **(A)** 340 ± 9, 338 ± 9, 333 ± 13, 312 ± 10, 310 ± 8, 304 ± 10, 307 ± 16 bpm in WKY and 394 ± 5, 360 ± 10^†^, 343 ± 13^†^, 325 ± 9^†^, 372 ± 6^†^, 328 ± 21^†^, 297 ± 5^†^ bpm in SHR (^†^; *P * ≤ 0.0083 compared to the controls), and in **(B)** 340 ± 9, 300 ± 8^†^, 318 ± 14 bpm in WKY and 394 ± 5, 333 ± 12^†^, 292 ± 7^†^ bpm in SHR (^†^*P * ≤ 0.004). * In brackets – *P * ≤ 0.05 after curve evaluations with Bonferroni-adjusted *P*-values.

**Figure 4:**
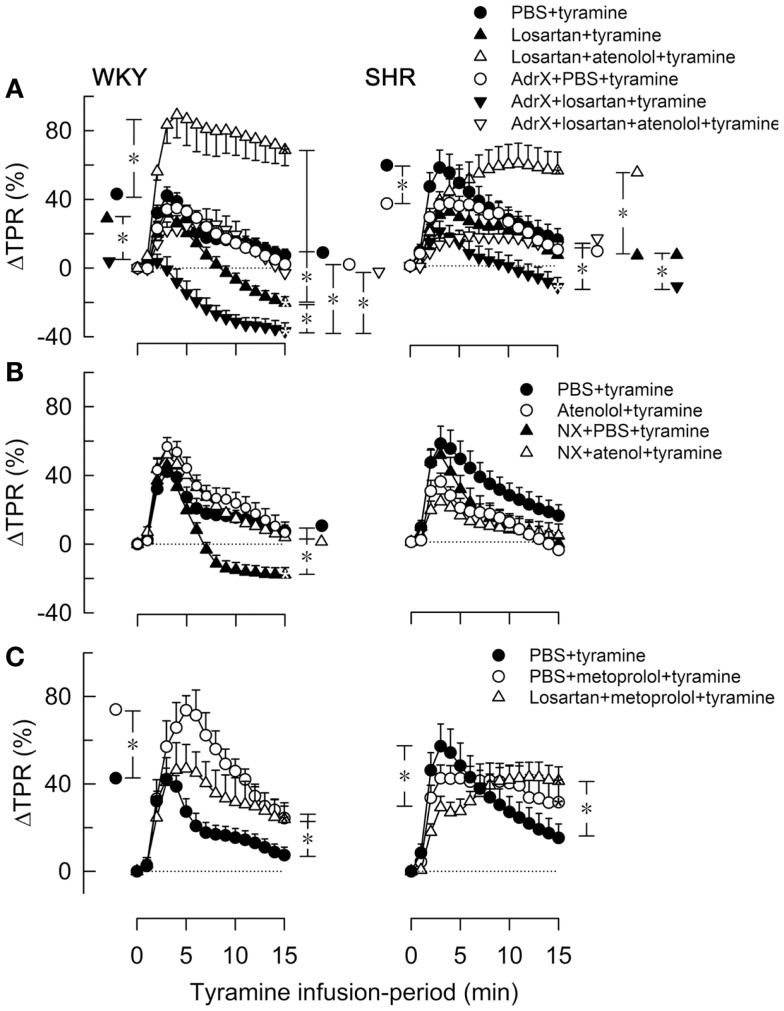
**The effect of atenolol (A, B) or metoprolol (C) on the TPR-response to tyramine in WKY and SHR**. The rats were pre-treated as outlined in the legends. After overall and step-by-step curve evaluations with Repeated Measures Analyses of Variance and Covariance (please see Statistical Analyses), significant responses were located at peak-response (all significant except in the WKY AdrX + losartan + tyramine group, not indicated) and at 15 min (* within symbol, as indicated). Group differences were located at the same times (* in brackets left and right of curves, respectively), as indicated. Comparisons were made in **(A)** between the PBS- and losartan-treated groups in not AdrX and AdrX rats, between the losartan- and AdrX + losartan-pre-treated groups, and between corresponding groups without and with pre-treatment with atenolol; in **(B)** between the controls and the NX groups, between corresponding groups without and with pre-treatment with atenolol; and in **(C)** between the controls and the metoprolol-treated groups, and between the metoprolol- and the losartan + metoprolol-treated groups. In the same order as in the legends, TPR baselines prior to tyramine was in mm Hg/mL/min in **(A)** 2.1 ± 0.1, 1.7 ± 0.1, 1.7 ± 0.1, 2.2 ± 0.1, 2.3 ± 0.2, 2.2 ± 0.2 in WKY and 4.8 ± 0.2, 4.0 ± 0.5, 4.1 ± 0.4, 6.3 ± 0.5, 7.6 ± 1.1, 5.1 ± 0.9 in SHR, in **(B)** 2.1 ± 0.1, 2.4 ± 0.1, 1.4 ± 0.1^†^, 1.9 ± 0.2 in WKY and 4.8 ± 0.2, 5.7 ± 0.4, 2.2 ± 0.3^†^, 4.1 ± 0.5 in SHR, and in **(C)** 2.1 ± 0.1, 2.0 ± 0.2, 1.6 ± 0.1^†^ in WKY and 4.8 ± 0.2, 4.3 ± 0.2, 4.1 ± 0.3 in SHR (^†^*P * ≤ Bonferroni-adjusted *P*-values). * In brackets – *P * ≤ 0.025 after curve evaluations with Bonferroni-adjusted *P*-values.

As previously documented (11), the tyramine-induced release of norepinephrine elicited a postsynaptic cardiovascular response, comprising a sustained increase in MBP, CO (not shown) and HR (Figure 3) and a transient rise in TPR (Figure 4).

The tyramine-induced tachycardia was reduced after atenolol and metoprolol (Figures 3A,B, respectively), showing efficient inhibition of cardiac, postsynaptic β_1_AR. The metoprolol-induced reduction in the tachycardia was eliminated in losartan-pre-treated SHR. Tyramine increased cardiac work load, and with a greater effect in WKY than SHR (Table 2). This increase was not significantly reduced by atenolol or metoprolol, alone or combined with losartan.

The losartan-dependant reduction in the TPR-response to tyramine, which was observed in WKY only, was further enhanced by AdrX (*P * ≤ 0.014), and a reduced ΔTPR was seen also in AdrX + losartan-pre-treated SHR (Figure 4A). Atenolol greatly increased the TPR-response in losartan-treated rats of both strains, also after AdrX (*P * ≤ 0.008), particularly in WKY (Figure 4A). Similar to that observed in losartan-treated rats, the late TPR-response to tyramine was reduced after NX in WKY only (*P * < 0.001), and this reduction was eliminated by atenolol (*P * = 0.002) (Figure 4B). Metoprolol alone increased the TPR-response to tyramine in WKY (*P * ≤ 0.007), particularly the early peak-response (Figure 4C). Pre-treatment with losartan + metoprolol elevated the late TPR-response in both strains (*P * ≤ 0.024 compared to the controls) (Figure 4C).

## Discussion

The main observation in the present study was that the β_1_AR subtype, like β_2_AR, functioned as a presynaptic, auto-receptor, and stimulated norepinephrine release from peripheral sympathetic nerves. However, neither β_1_- nor β_2_AR influenced the secretion of epinephrine. In addition, atenolol combined with losartan greatly increased the rise in TPR in response to endogenous norepinephrine release.

### Use of tyramine to study presynaptic modulation of norepinephrine release *in vivo*

Norepinephrine overflow to plasma is normally low due to synaptic re-uptake through NET (Figure 1), and presynaptic control of basal release from presynaptic vesicles has little influence on the plasma norepinephrine concentration in the anesthetized rat (9). Indeed, the β_1+2_AR antagonist nadolol did not alter norepinephrine overflow to plasma even during stimulated, vesicular norepinephrine release, sufficient to cause a maximum increase in HR (18). However, when re-uptake through NET was blocked by desipramine, the inhibiting effect of presynaptic α_2_AR on release (Figure 1) could be demonstrated by an α_2_AR antagonist as an increase in norepinephrine overflow, even without stimulation of release (9). Reversed transport of norepinephrine through NET can be stimulated by tyramine (Figure 1). This transport is not under presynaptic control (19). However, by engaging NET in release, tyramine prevents re-uptake, and also supplies norepinephrine as agonist for presynaptic facilitation and inhibition of a concomitant vesicular release. Presynaptic stimulation and inhibition of vesicular release is therefore reflected as differences in the tyramine-induced norepinephrine overflow to plasma (6, 10, 11). The questions posed in the present investigation, involved several organs, and could therefore be answered only through experiments on intact animals. Tyramine-induced norepinephrine overflow to plasma provided a method to study how other organs and systems may contribute to the control of norepinephrine release.

### Demonstration of β_1_AR as a presynaptic auto-receptor, facilitating the release of norepinephrine

The present results clearly demonstrated that not only the β_2_AR-selective antagonist ICI-118551, but also the β_1_AR-selective antagonists CGP20712A, atenolol and metoprolol lowered tyramine-induced norepinephrine overflow. Since tyramine and atenolol do not readily cross the blood–brain barrier, it was concluded that the β_1_AR involved was peripherally located. The effect of the β_1_AR antagonists was slightly greater than that of the β_2_AR antagonist in WKY, but not in SHR. However, inhibition of both β_1_- and β_2_AR did not lower overflow more than β_1_- or β_2_AR antagonist alone in either strain. This result demonstrated that the effect of the two βAR was not additive, compatible with a common signaling pathway for both receptors, i.e., activation of adenylyl cyclase (Figure 1).

The catecholamine responsible for the β_1_- and β_2_AR-mediated stimulation of norepinephrine release did not rely on catecholamines secreted from the adrenals during the experiment. This was concluded from the fact that β_1_- or β_2_AR antagonist reduced tyramine-induced norepinephrine overflow also in AdrX rats. However, the adrenals were removed only 30 min prior to administration of antagonist. Epinephrine, previously taken up from the circulation through NET, may therefore still be present in the nerve terminal vesicles and co-released with norepinephrine (5). Since the affinity of the β_2_AR is much higher for epinephrine than norepinephrine, it could not be excluded that this epinephrine activated presynaptic β_2_AR. On the other hand, the affinity of the β_1_AR for norepinephrine and epinephrine does not differ. It was therefore concluded that norepinephrine, released by tyramine from peripheral nerves, activated the peripheral β_1_AR which facilitated norepinephrine release. Atenolol in addition reduced tyramine-induced norepinephrine overflow after pre-treatment with AdrX + hexamethonium in both strains. Thus, the reduced overflow of norepinephrine after pre-treatment with atenolol also did not involve a central nervous system component or β_1_AR-mediated stimulation of ganglion transmission.

The slightly reduced tyramine-induced norepinephrine overflow after losartan in WKY was likely to result from inhibition of presynaptic AT1R, which stimulate release (13, 20). However, losartan did not eliminate the hampering effect of atenolol or metoprolol on norepinephrine overflow in WKY or SHR, and atenolol reduced tyramine-induced norepinephrine overflow also in 24-h NX rats. It was therefore concluded that the stimulating effect of β_1_AR on norepinephrine release was not mediated through activation of renal renin secretion (21), with subsequent activation of presynaptic, release-stimulating AT1R (Figure 1). Atenolol reduced norepinephrine release also in AdrX + losartan-pre-treated WKY. AT1R-mediated stimulation of adrenal catecholamines (22) was therefore not involved. However, atenolol did not lower norepinephrine overflow in AdrX + losartan-pre-treated SHR. This observation may possibly be explained by the fact that AT1R antagonist restored α_2_AR-mediated inhibition of norepinephrine release in this strain (11). A losartan-dependent, enhanced α_2_AR-mediated inhibition of release may therefore possibly counter-act the effect of β_1_AR-mediated stimulation and, hence, the effect of atenolol, when also a substituting, β_2_AR-mediated stimulation of release was eliminated by AdrX (Figure 1).

Hexamethonium itself reduced tyramine-induced norepinephrine overflow in SHR, possibly explained by inhibition of presynaptic, nicotinic receptors, which stimulated vesicular release (23). But it may also result from inhibition of ganglion transmission, which will lower the sympathetic nerve tone, which is responsible for the vesicular release. The latter possibility was supported by that hexamethonium reduced MBP, HR, TPR, and cardiac work load with a greater effect in SHR than in WKY. However, in WKY, and even more in AdrX SHR, hexamethonium enhanced norepinephrine overflow. The mechanism underlying this observation was not clarified by the present experiments.

From these experiments, it was concluded that β_1_AR functioned as a presynaptic auto-receptor in peripheral sympathetic nerves and stimulated norepinephrine release. This conclusion differed from that previously observed in nerve-stimulated rat portal veins and human arteries and veins where β_2_AR alone enhanced norepinephrine release (3, 4, 24). However, both subtypes enhanced electrically stimulated norepinephrine release in rat brain slices and canine intrathoracic ganglia (8, 25), and β_1_AR mRNA and protein have been detected in rat sympathetic neuronal cell bodies and axons (26). The present results were also fully compatible with the increased ventricular sympathetic axon proliferation in rats given a 1-week treatment with metoprolol (26), possibly to compensate for a reduced norepinephrine release. Tyramine selectively stimulates norepinephrine release through NET, whereas electrical stimulation elicits release from the nerve terminal vesicles, which may contain also other transmitters, including epinephrine. Since presynaptic control of release regulates vesicular release only (19), β_1_AR-inhibition will lead to a down-regulation of release of transmitters other than norepinephrine, while still permitting NET-mediated norepinephrine release. The present exclusive release of norepinephrine stimulated by tyramine may therefore favor the demonstration of the facilitating β_1_AR.

### Control of adrenal epinephrine secretion

Tyramine did not evoke secretion of epinephrine, and the elevated plasma concentration was due to the stress-induced by the surgery (9, 10). This was as expected, since adrenal cortical glucocorticoid release with subsequent epinephrine secretion may respond to any type of stress, including surgery, and is not, like norepinephrine release from the adrenal and most sympathetic nerves, regulated by the baroreceptor reflex. Epinephrine secretion is mediated through nicotinic receptors on adrenal chromaffin cells (27); in accordance with the observed clear reduction after pre-treatment with the nicotinic receptor antagonist hexamethonium in both strains. The plasma epinephrine concentration was not reduced after any of the βAR antagonists in either strain, in spite of that inhibition of the α_2A_AR increased epinephrine secretion in similar experiments (9). Although losartan through a central action may lower stress-induced sympathoadrenal activation (17), losartan did not alter the concentration of epinephrine. However, the plasma epinephrine concentration was higher in 24-h NX WKY but not SHR. It may be speculated that this increase resulted from removal of afferent renal nerve signaling, which lowers sympathetic output, at least to the kidney (28). This reflex was reduced in SHR (29). The mechanism may involve peripheral β_1_AR, since the increased epinephrine concentration in NX WKY was eliminated by atenolol.

### Implications of presynaptic, stimulating β_1_AR in the treatment of hypertension

Atenolol is the most commonly used β_1_AR-blocker in the treatment of hypertension. Hypertensive disease is in most patients due to increased sympathetic nerve activity (30). This is likely to be due to an increased central sympathetic output, since clonidine reduced resting norepinephrine overflow, MBP, HR, and TPR through a central action in SHR but not WKY (9). The present results indicated that the antihypertensive effect of β_1_AR-selective antagonists may be explained by their ability to hamper norepinephrine release through presynaptic modulation, in agreement with previous analyses showing that the hypotensive effect of AR antagonists, including atenolol, did not depend on cardio depression or suppression of renin secretion (31). Sympathetic nerve activation is also seen in heart failure and myocardial infarction, first compensating for the failing function, but eventually it has a detrimental effect, causing receptor desensitization due to long-term exposure to increased levels of norepinephrine. The beneficial therapeutic effect of β_1_AR-selective antagonists in such cardiac conditions may therefore include also their ability to lower norepinephrine release.

The two β_1_AR-selective antagonists atenolol and metoprolol, when injected IV, reduced resting HR but had little effect on TPR, and only atenolol caused a minor reduction in MBP and in SHR only, due to a reduction in CO. Since the ganglion blocker hexamethonium reduced all cardiovascular parameters in this strain, including TPR, the acute effect of the β_1_AR antagonists was primarily cardio-selective and due to postsynaptic intervention. As expected from the elevated sympathetic tone in SHR, atenolol and metoprolol reduced resting HR, and atenolol, but not metoprolol, lowered resting cardiac work load with a greater effect in SHR than in WKY. A reduced work load was seen also after losartan, with a further reduction after addition of atenolol or metoprolol in SHR only. These effects were compatible with reduced cardiac energy consumption, which is a therapeutic goal in heart failure and myocardial infarction.

### Possible cardiovascular complications resulting from the use of β_1_AR antagonists

The tyramine-induced tachycardia was not prevented by metoprolol when combined with losartan in SHR, and atenolol and metoprolol, alone or combined with losartan, had little effect on the tyramine-stimulated rise in cardiac work load in either strain. The transient rise in TPR during tyramine-induced norepinephrine release was, as previously described (11), changed to vasodilatation during the late part of the tyramine infusion in losartan-treated WKY but not in SHR. This observation indicated, as expected, that angiotensin II-AT1R-activity may potentiate norepinephrine-induced vasoconstriction. The same pattern was observed in NX rats, indicating that the angiotensin II-formation depended on renin released from the kidneys. Furthermore, after AdrX + losartan, the TPR-peak-response was eliminated in WKY, and was reduced throughout the tyramine infusion-period in SHR. These observations were not explained by differences in norepinephrine release, and apparently indicated increased postsynaptic, adrenal catecholamine-dependent control of vascular tension in the absence of AT1R-activity. However, in the presence of losartan, atenolol greatly enhanced the TPR- and MBP-response to tyramine, particularly in WKY and also in AdrX rats of both strains. It therefore appeared that VSMC β_1_AR-mediated vasodilatation played a crucial role in antagonizing AT1R-mediated vasoconstriction. This pattern differed from that observed after pre-treatment with metoprolol. Metoprolol increased the TPR-peak-response to tyramine in WKY, but not when combined with losartan, whereas metoprolol increased the late TPR-response in SHR only if combined with losartan. This difference may be due to that metoprolol, unlike atenolol, readily crosses the blood–brain barrier. These observations may be of great clinical importance. Norepinephrine is normally released from nerve terminal vesicles in response to sympathetic nerve action potentials, and is lowered through presynaptic receptors by the β_1_AR antagonists. However, myocardial ischemia lasting more than 10 min will cause nerve terminal ATP depletion, hypoxia, and a fall in intracellular pH, leading to massive, reverse transport through NET, with extracellular catecholamine accumulation up to 100–1000 times greater than that in plasma (32). Thus, during local or global cardiac ischemia, the condition will be comparable to that induced by tyramine-stimulation, and inhibition of β_1_AR-mediated vasodilatation may disturb cardiac perfusion, particularly if combined with losartan, without having the desired effect on cardiac work load and cardiac energy consumption. This may explain why vasodilatory β-blockers reduced overall mortality with a greater effect than non-vasodilatory β-blockers such as atenolol (33).

## Conclusion

Peripheral β_1_AR, like β_2_AR, were for the first time demonstrated *in vivo* to function as a presynaptic, auto-receptor, facilitating release of norepinephrine from peripheral sympathetic nerves in WKY and SHR. Inhibition of norepinephrine release may therefore explain the antihypertensive effect of β_1_AR-selective antagonists such as atenolol, the most frequently used β-blocker in the treatment of hypertension. The reduced norepinephrine release may be beneficial also in other conditions with sympathetic hyperactivity such as heart failure and myocardial infarction in addition to the sparing effect of β_1_-blockers on cardiac work load and energy consumption. However, when massive norepinephrine release was precipitated by reverse transport through NET, as by tyramine in the present study, but which may be induced by hypoxia during myocardial infarction and ischemic heart disease, the sparing effect of the β-blockers on cardiac work load was limited. In addition, inhibition of postsynaptic, β_1_AR-mediated vasodilatation greatly increased norepinephrine-induced vasoconstriction. This response may hamper organ perfusion, and aggravate the condition they are meant to prevent. This reaction was particularly pronounced when atenolol was combined with losartan, a commonly used drug combination in the clinic. The role of reversed NET transport of norepinephrine in cardiovascular events may therefore deserve more attention.

## Conflict of Interest Statement

The author declares that the research was conducted in the absence of any commercial or financial relationships that could be construed as a potential conflict of interest.
